# Large-scale transfer of Ag nanowires from PET to PC film using a roll-to-roll UV lamination process for a capacitive touch sensor

**DOI:** 10.1039/d2ra05600c

**Published:** 2023-01-06

**Authors:** Yangkyu Park, Jae Pil Kim, Wan Ho Kim, Young Hyun Song, Sunyoon Kim, Ho-Jung Jeong

**Affiliations:** a Department of Mechanical Design Engineering, Chonnam National University 50 Daehak-ro Yeosu Chonnam 59626 Republic of Korea; b Corporate Growth Support Center, Jeonnam Yeosu Industry-University Convergence Agency 17 Samdong 3-gil Yeosu Chonnam 59631 Repulic of Korea; c Lighting Materials and Components Research Center, Korea Photonics Technology Institute (KOPTI) Gwangju 61007 Republic of Korea hojung@kopti.re.kr

## Abstract

Demand for flexible transparent sensors for futuristic cars is increasing since such sensors can enhance the freedom of design and aesthetic value in the interior of cars. Herein, we propose a unique roll-to-roll UV lamination process that can expedite large-scale Ag nanowire (AgNW) transfer for a flexible capacitive sensor, using a photocurable resin composed of an epoxy acrylate oligomer, a reactive monomer (1,6-hexanediol diacrylate), and a photoinitiator (1-hydroxycyclohexyl phenyl ketone). The acryl groups in the resin were rapidly crosslinked by UV irradiation, which facilitated the AgNWs transfer from a PET to a PC substrate with the speed of 1050 cm^2^ min^−1^ and enhanced the adhesion between the AgNWs and the PC substrate. Systematic experiments were performed to determine optimal fabrication parameters with respect to the UV dose, lamination pressure, and laser dicing conditions. At the optimal fabrication conditions, the sheet resistance of AgNWs on a PC film (PC-AgNW) was as small as 36.79 Ω sq^−1^, which was only 3.17% deviation from that on a PET film (PET-AgNW). Furthermore, the optical transmittance of the PC-AgNW exceeded 88% over the visible range, and it was greater than that of the PET-AgNW. Notably, the sheet resistance of the PC-AgNW was almost constant after 50 taping and peeling cycles, indicating remarkable adhesion to the substrate. Furthermore, a capacitive touch sensor was fabricated using the PC-AgNW, and its switching signals were presented with and without finger touch.

## Abstract 2

We proposed a unique roll-to-roll lamination process that can facilitate the transfer of Ag nanowires (AgNWs) from one flexible substrate (PET substrate) to another (PC substrate) for a capacitive touch sensor.

## Graphical abstract

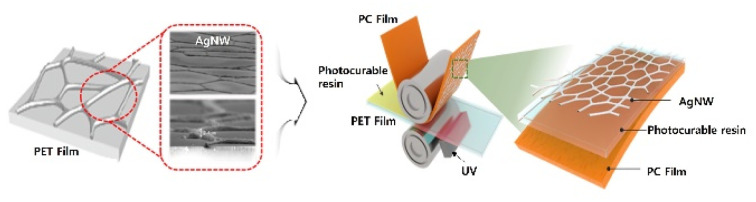

## Introduction

With the increasing importance of the safety and aesthetics of cars, demand for infotainment systems is increasing in futuristic cars. The existing car interior has evolved, and residential interior for “living spaces on wheels” has been introduced.

An integrated display with many digital buttons improves the aesthetics and freedom of car interior design. However, multiple user interfaces integrated within a limited display space cause inconvenience since they increase the number of touches by the driver. To minimize the number of touches, car manufacturers should position digital buttons directly on the complex 3D surfaces of car interiors instead of integrating them into the display. This will allow the existing interior parts to be reproduced as aesthetic electronic machines.

For the realization of touch sensors in futuristic cars, the sensor electrodes should have high mechanical stretchability, optical transmittance, and electrical conductivity. Indium tin oxide (ITO) has been commonly used as the electrode. However, its ceramic nature leads to brittle characteristics that cause irreversible loss of electrical conductivity at strains above 1%.^1–4^ Representative substitutes for the ITO are carbon-based materials containing carbon nanotubes or graphene, conductive polymers such as poly(3,4-ethylenedioxythiophene):poly(styrenesulfonate) (PEDOT:PSS), and metal nanowires. Carbon nanotubes have relatively low transmittance, conductivity, and difficulties in separation, purification, and dispersion from the raw material.^3,5^ Graphene has superior intrinsic properties; however, large volume production could be challenging. Conductive polymers are known to lose their conductivity when exposed to high humidity, temperature, or ultraviolet (UV) light.^3,5^ Metal nanowires are promising alternatives to the ITO as transparent flexible electrodes.^1,6–9^ Although the high electron density in metals could make them opaque, metal nanowires smaller than visible wavelength can be highly transparent while having good conductivity. Among several metal wires, AgNWs show outstanding properties: a low sheet resistance of 36 Ω sq^−1^ and a high optical transmittance of 97.9% at 550 nm.^10^

Despite their good properties, the weak adhesive forces between AgNWs and plastics are a critical issue restricting the practical use of AgNWs.^11,12^ Common methods used to overcome this limitation is coating AgNWs with transparent conducting materials such as graphene,^13^ ITO,^14^ and ZnO.^15^ However, the complex processing conditions could lead to high production costs; the conducting oxide layers are intrinsically brittle, which restricts the mechanical flexibility. Recently, the use of hot lamination techniques for transferring graphene or AgNWs to flexible substrate slices has been investigated and have led to good adhesion in a simple and cost-effective way.^11,16^

Polyethylene terephthalate (PET) is the most common flexible film used for AgNW substrates.^17–19^ However, PET is highly susceptible to UV light.^20,21^ Photo-oxidation and hydrolytic cleavage initiated by UV light are common under ambient conditions.^21^ PET degradation can result in discoloration (yellowing) and/or haze formation.^20,21^ Furthermore, the transition temperature of PET is relatively low (67–81 °C), and hence, it is easily affected by the temperature. To readily adapt flexible electronics to outdoor applications, flexible substrates with better UV and thermal resistance, such as a polycarbonate (PC), would be preferable.

In this study, for the fast large-scale fabrication of a AgNW film in which AgNWs have strong adhesion to flexible substrates, we developed a unique roll-to-roll UV lamination process. Unlike the previous hot lamination processes,^11,16^ our lamination involves a photocurable resin as a bonding layer between electrodes and the target substrate, and this method is applied to a roll-to-roll process. In contrast to previous roll-to-roll slot die coatings that directly deposit a conductive liquid onto a target substrate surface,^12,22^ we propose AgNW transfer from a PET to a PC substate for effective electrode transfer. The poor adhesion of AgNWs to PET allows the electrodes to be easily transferred to the PC substrate through the bonding layer. The UV exposure dose, lamination pressure, and laser dicing power/speed were examined to determine the optimal fabrication conditions. The electrical, optical characteristics, and adhesion to the substrates were estimated. Furthermore, a capacitive touch sensor was fabricated using our PC-based AgNW, and the sensor responses are presented with and without finger touch.

## Experiments

### Materials and methods

**Fig. 1:**
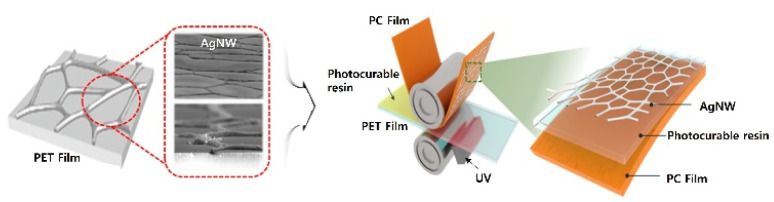
Schematic of the roll-to-roll lamination process for transferring AgNW from a PET film to a PC film.

Fig. 1 depicts our roll-to-roll UV lamination process; AgNWs were prepared on a PET film (PET-AgNW), and the AgNWs were then transferred to a PC film (PC-AgNW) through a photocurable resin. The raw materials used for preparing the PET-AgNW included AgNW (NovaWire-Ag-A60, Novarials, USA), toluene (Sigma-Aldrich, USA), decamethylcyclopentasiloxane (DMCPS; Sigma-Aldrich, USA), 2-amino-3-methyl-1-butanol (AMB; Sigma-Aldrich, USA), poly-ethersiloxane (PES; Sigma-Aldrich, USA), hexamethoxymethylmelamine (HMMM; Sigma-Aldrich, USA), dinonylnaphthalene disulfonic acid (DNNDSA; Sigma-Aldrich, USA), distilled deionized water (DDW), and PET film (V7610, SKC, Republic of Korea). Also, PC film (Sabic, Saudi Arabia) and a photocurable resin (QF1818, QENTOP, Republic of Korea) comprising an epoxy acrylate oligomer, 1,6-hexanediol diacrylate for a reactive monomer, and a 1-hydroxycyclohexyl phenyl ketone as photoinitiator were used for the PC-AgNW. The photocurable resin features high transmittance and it was used as an acylate-based adhesive between AgNWs and the PC film. The acryl groups were crosslinked by UV exposure for fast large-scale transfer of the AgNWs with strong adhesion property.

### Roll-to-roll lamination setup

**Fig. 2:**
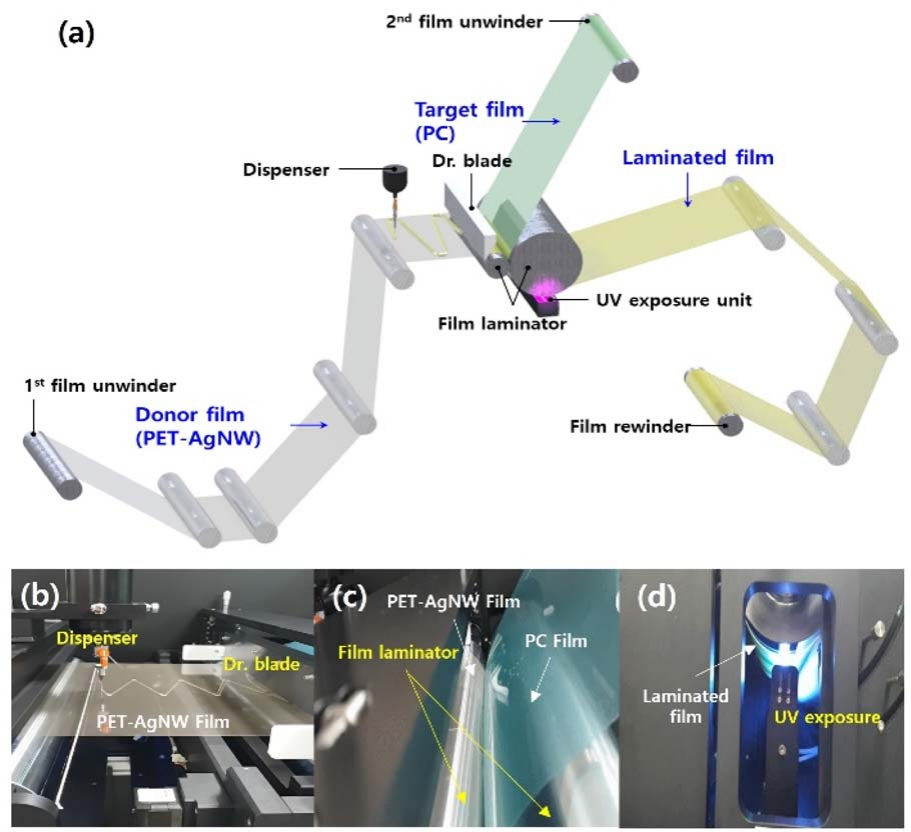
Roll-to-roll UV lamination setup. (a) Overall schematic view and photographs of the (b) dispenser, Dr blade, (c) film laminator, and (d) UV exposure unit.

As shown in Fig. 2(a), the roll-to-roll machine comprised two film unwinders, a dispenser, Dr blade, a film laminator, a UV exposure unit, and a film rewinder. Fig. 2(b–d) shows photographs of the dispenser, Dr blade, film laminator, and UV exposure unit. The first film unwinder supplied a donor film binding the electrodes to the coater. As depicted in Fig. 2(b), a dispenser in the coater spaced apart from the donor film coats the photocurable resin in a zigzag pattern. The resin on the donor film is spread with a uniform thickness by a Dr blade, and the donor film is supplied to the laminator. The second film unwinder provides the laminator with a target film to be positioned on the resin layer of the donor film. The laminator is located adjacent to the Dr blade and consists of two rollers, as shown in Fig. 2(c). The two rollers laminate the donor and target film with the resin by applying a constant pressure. A laminated film is supplied to the UV exposure unit, which is located below the laminator, and the resin is cured through UV exposure, as depicted in Fig. 2(d). The rewinder at the rearmost end of the roll-to-roll machine winds the laminated film.

### PET-AgNW fabrication

An amount of 2 g of AgNWs and 58 g of toluene were stirred at 10 000 rpm for 1 h in a magnetic stirrer to prepare 60 g of an AgNW dispersion. A mixture of DMCPS (0.35 g), AMB (0.35 g), and PES (0.4 g) was prepared for use as an additive for increasing wettability and dispersion stability of the AgNW dispersion.^23^ Subsequently, HMMM (0.15 g), DNNDSA (0.15 g), and the additive (1.1 g) were added sequentially to 38.6 g of DDW to prepare 40 g of an aqueous dispersion. The AgNW dispersion (60 g) and the aqueous dispersion (40 g) were stirred overnight at 10 000 rpm in the magnetic stirrer to obtain 100 g of AgNW coating solution. The prepared AgNW coating solution was uniformly coated on a PET film with an area of 350 mm × 300 mm and a thickness of 27.5 μm by using an automatic film coater (AP-P100, AP Lab, Republic of Korea), and it was heated for 1 h at 50 °C. Subsequently, PET-AgNW was fabricated with a 7 μm-thick electrode.

### PC-AgNW fabrication

The prepared PET-AgNW wound on a paper tube with an inner diameter of 76.2 mm and a width of 500 mm was supplied to the coater by the first film unwinder. The photocurable resin was coated on the PET-AgNW film by the dispenser, and the coating thickness was controlled to be 15 μm by the Dr blade. The PET-AgNW film and PC film were supplied to the film laminator at the same velocity of 30 mm s^−1^. The two films were laminated under various pressures to investigate the electrode transfer characteristics with respect to the applied pressures. The operation of the entire rollers temporarily stopped during UV exposure. The electrode transfer features with respect to the UV exposure doses were examined by adjusting the exposure time at the UV (365 nm, 1 W) exposure unit. After film rewinding, the PET film was delicately detached from the PC-AgNW. Subsequently, PC-AgNW was laser diced by a femtosecond laser (SM-LMM-9100, SMTECH, Korea) at 343 nm. The laser power and the speed were regulated to find the optimal dicing conditions.

### Characterization of PET-AgNW and PC-AgNW

The degree of curing of the resin with respect to the exposure dose was investigated using Fourier transform infrared (FTIR) spectrometry (Spotlight 400, PerkinElmer, USA). The sheet resistances of the PET-AgNW and PC-AgNW were measured using a four-probe-based resistivity meter (MCP-T610, Mitsubishi Chemical Analytech, Japan), and at least 10 measurements were obtained at arbitrary points to verify the accuracy of data. An optical microscope (SMZ 1000, Nikon, Japan) and a scanning electron microscope (SEM; SU8010, Hitachi, Japan) were used to analyze the mesh shapes of the AgNWs formed on the PET and PC films. The optical transmittances of the PET-AgNW and PC-AgNW were measured by a UV-Vis-NIR spectrophotometer (Cary 5000, Varian, USA) in the range from 300 to 800 nm. Adhesion tests were conducted using 3 M Scotch 810 tape.

## Results and discussion

To determine the optimal fabrication parameters for the PC-AgNW, we conducted systematic experiments by considering the (1) UV exposure time, (2) lamination pressure, and (3) laser dicing conditions.

**Fig. 3:**
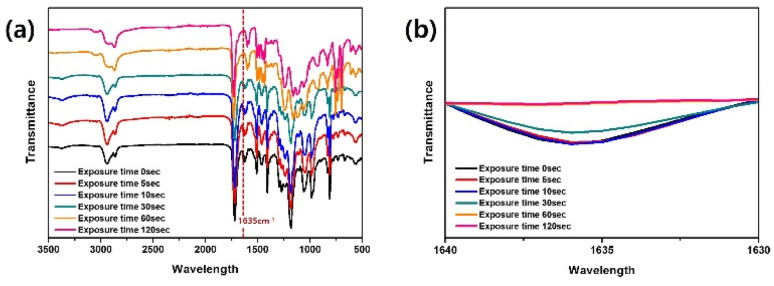
FTIR spectra with respect to the exposure time at (a) the full measurement range (3500 cm^−1^ to 500 cm^−1^) and (b) a specific range where a distinct peak was observed (1640 cm^−1^ to 1630 cm^−1^).

FTIR analysis was performed to estimate the degree of curing of the resin. Specifically, the PC-AgNW was laser diced into 1 cm × 1 cm pieces, and the curing behavior was analyzed by observing peak changes at 1635 cm^−1^, which is associated with the C=C stretching vibration.^24^ The exposure time was increased from 0 to 120 s, which corresponded to a UV dose of 240 mJ cm^−2^. As shown in Fig. 3, the peak centered at 1635 cm^−1^ increased with the exposure time, confirming the occurrence of polymerization. A significant change at 1635 cm^−1^ was not observed when the exposure time exceeded 60 s, which corresponded to 120 mJ cm^−2^.

**Fig. 4:**
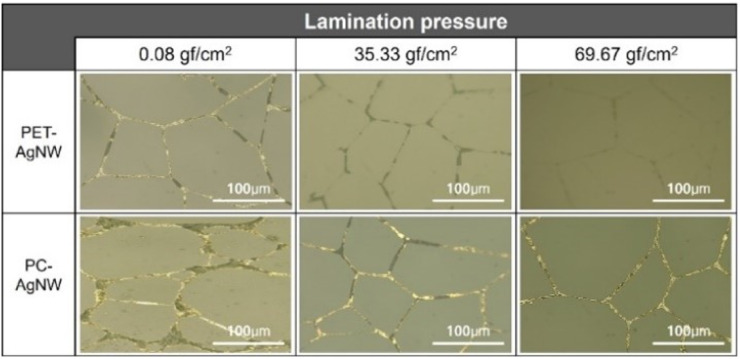
Electrode transfer comparison at different lamination pressures. The two rows represent PET-AgNW and PC-AgNW, and the columns represent lamination pressures of 0.08, 35.33, and 69.67 gf cm^−2^.

Fig. 4 shows the fabrication results for the PET-AgNW and PC-AgNW before and after electrode transfer, respectively, with respect to the lamination pressure for 60 s of UV exposure. The resin thickness between the PET and PC films decreased from 20.1 μm to 9.5 μm as the lamination pressure increased from 0.08 gf cm^−2^ to 69.67 gf cm^−2^. Notably, as the pressure increased, the rate of electrode transfer increased, as shown in Fig. 4. This indicates that the higher the applied pressure, the better the adhesion through the resin. Thus, the lamination contributed significantly to the adhesion.

**Fig. 5:**
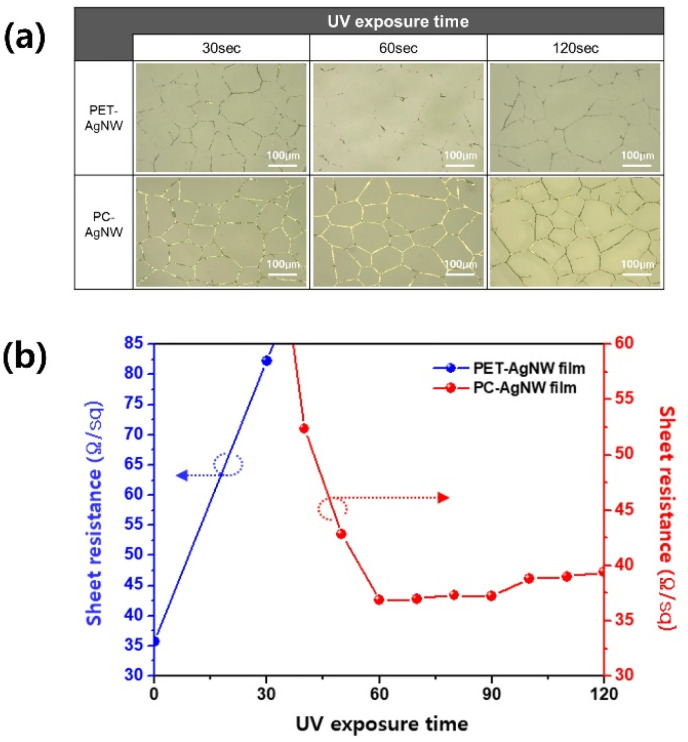
Fabrication results of PET-AgNW before electrode transfer and PC-AgNW after electrode transfer at different UV curing times. (a) Optical microscope images and (b) sheet resistance changes.

Fig. 5 shows optical microscope images of the PET-AgNW before electrode transfer and of the PC-AgNW after electrode transfer. When the UV exposure time was less than 60 s, the electrodes remaining on the PET film were clearly visible. By contrast, large portions of the electrodes were transferred to the PC-film at 60 and 120 s. The rate of change of the sheet resistance of the PC-AgNW was negligible at exposure times exceeding 60 s, which agreed with the results of FTIR analysis.

For 60 s of UV exposure time and 69.67 gf cm^−2^ lamination pressure, the measured sheet resistance of the PC-AgNW was as small as 36.79 Ω sq^−1^. Compared with the values of 35.66 Ω sq^−1^ for the PET-AgNW, the deviation in the sheet resistance was estimated to be only 3.17%.

**Fig. 6:**
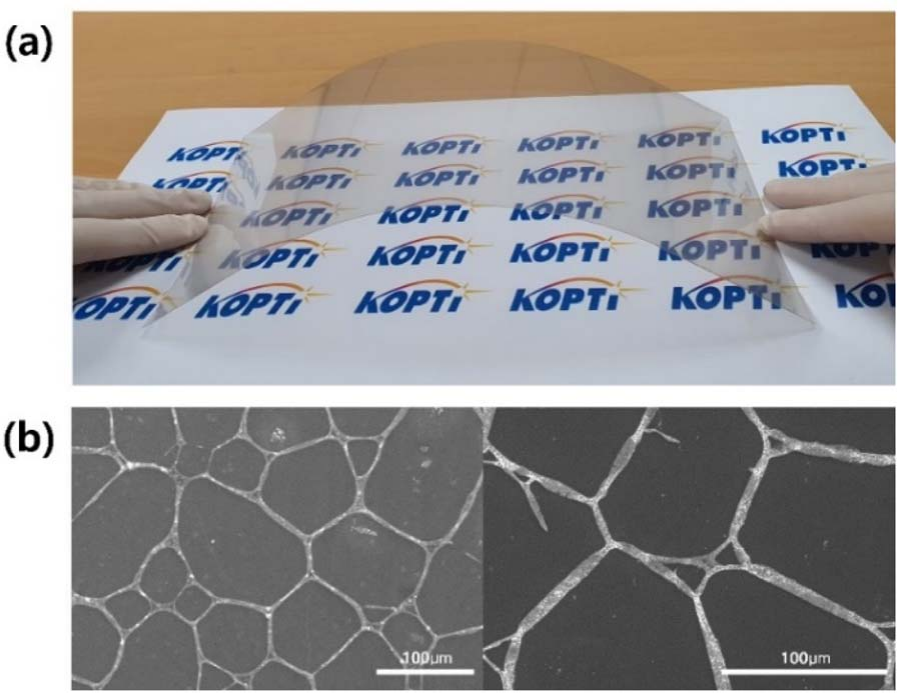
PC-AgNW fabricated by our roll-to-roll UV lamination process. (a) Digital camera image of the highly transparent flexible electrode of PC-AgNW and (b) SEM images with different magnifications.

The electrode transfer of the AgNW could be confirmed from the SEM images shown in Fig. 6. Considering the electrode area of the PET-AgNW (350 × 300 mm^2^) and the optimal UV exposure time (60 s), we estimated the speed of electrode transfer between the PET film and the PC film to be 1050 cm^2^ min^−1^, indicating fast large-scale electrode transfer.

Compared to the hot lamination process^11^ using thermal heating to enhance the adhesion of the nanomaterial, our roll-to-roll UV lamination process utilized the photocurable resin. Thereby, electrode transfer speed was much faster for the UV lamination process (1050 cm^2^ min^−1^) than the hot lamination process (2 min for one substrate slice).^11^

The optimal laser dicing conditions for forming a flexible electrode of a desired shape without any damage were estimated. The target shape of the electrode was set to be a circle with a diameter of 0.5 cm. After the dicing of the electrode, the open/short connections between the inside and outside of the circle were measured by a digital multimeter. The laser power and speed were controlled from 0.15 W to 0.55 W and from 5 mm s^−1^ to 30 mm s^−1^, respectively.

**Fig. 7:**
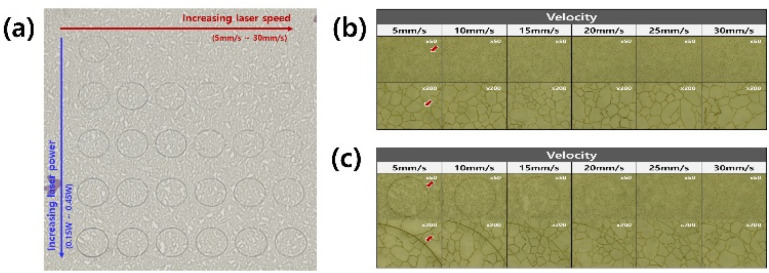
Experimental results of laser dicing with different laser powers and laser speeds. (a) A digital camera image of a test PC-AgNW film on an A4 paper. The diced patterns toward the right columns (direction of red arrow) and in the lower row (direction of blue arrow) were fabricated with higher moving speeds and higher powers, respectively. Optical microscope images of the electrodes diced under (b) 0.15 W and (c) 0.45 W at laser speeds of 5, 10, 15, 20, 25, and 30 mm s^−1^.

Fig. 7 shows optical microscope images of the electrode diced under 0.15 W and 0.45 W at different speeds of a laser head. A clear circular shape and an electrical open connection were confirmed at 0.45 W and 5 mm s^−1^. On the other hand, an electrical short connection was measured when the laser power was less than 0.4 W or the moving speed exceeded 10 mm s^−1^. The PC-AgNW was burnt occasionally during laser dicing with a power exceeding 0.5 W.

**Fig. 8:**
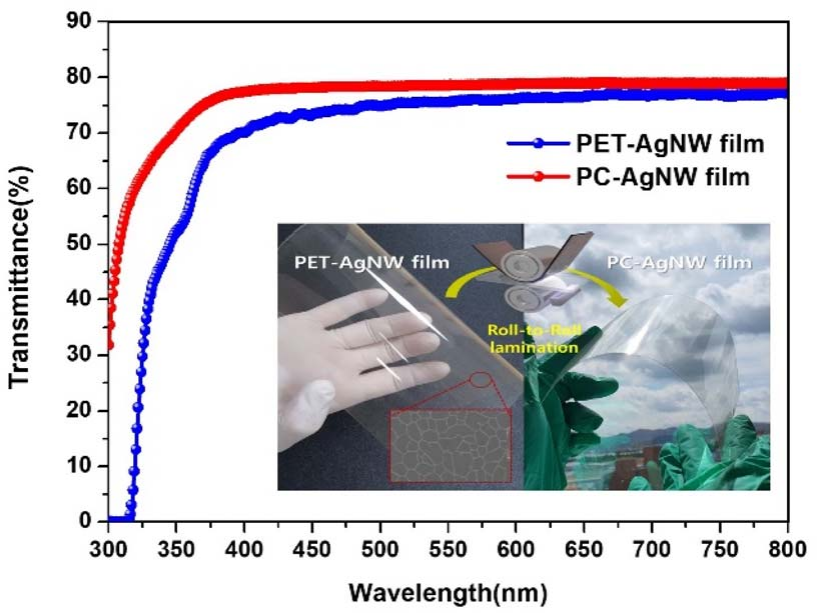
Optical transmittance measurements of PET-AgNW and PC-AgNW. The transmittance of PC-AgNW after electrode transfer was improved to a value higher than that of PET-AgNW before electrode transfer. The insets show digital camera images of PET-AgNW and PC-AgNW.

The optical transmittances of the PET-AgNW and PC-AgNW were measured from 300 nm to 800 nm. The transmittance of the PC-AgNW was above 88% over the visible range, as shown in Fig. 8. It is noteworthy that the PC-AgNW transmittance was more enhanced than the PET-AgNW transmittance because the transmittance of the PC film is better than that of the PET film. This implies that if our roll-to-roll lamination process were to be applied to other functional films with anti-reflection property, UV shield, anti-glare property, high haze, *etc.*, superior performance could be achieved with large-scale manufacturing. In order to evaluate the properties of flexible electrodes, the ratio of direct-current (DC) conductivity to optical conductivity, *σ*_DC_/*σ*_OP_ was used, and it is shown in eqn (1); *σ*_DC_, *σ*_OP_, *R*_sh_, and *T*(*λ*) are the DC conductivity, optical conductivity, sheet resistance, and optical transmittance at 550 nm wavelength, respectively.^2,22^1
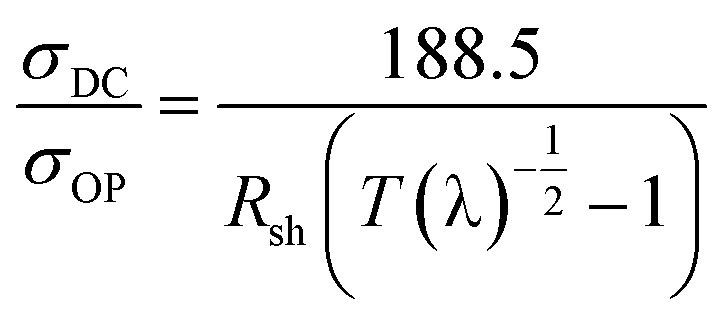


The equation suggests that an electrode with higher conductivity and yet low optical absorbance corresponds to better transparent electrodes. The ratio (*σ*_DC_/*σ*_OP_) of our PC-AgNW was estimated as 79, which was higher than the value 71 of the PET-AgNW and much higher than the reference value of 35 for the use in practical devices.^22^

**Fig. 9:**
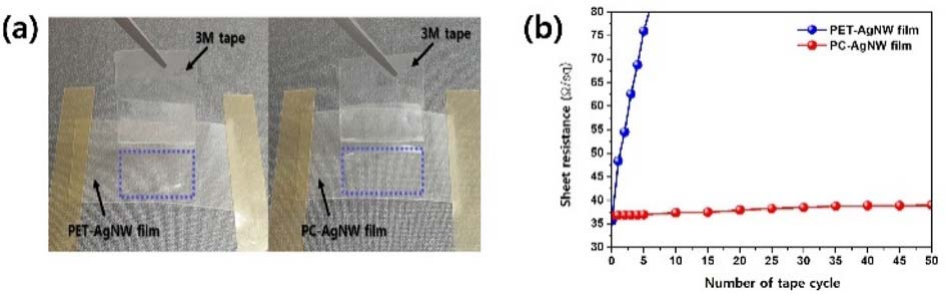
Adhesion test results. (a) Test images of the PET-AgNW and PC-AgNW. The blue rectangles show the areas where the peeling tests were performed. (b) Sheet resistance variation as a function of the number of taping and peeling cycles.

It is known that AgNWs without any treatment do not stick well to the substrate and can be easily wiped off by a tape. To measure the AgNW adhesion quantitatively, the sheet resistance variations were monitored through several taping and peeling cycles, as depicted in Fig. 9. Indeed, the PET-AgNW lost its conductivity after 5 cycles, whereas obvious variations were not measured even after 50 cycles, implying that most of the AgNWs on the PC stay in their original positions because of the laminated resin. Thus, this result demonstrated the remarkable adhesion enhancement achieved by using our roll-to-roll UV lamination process.

**Fig. 10:**
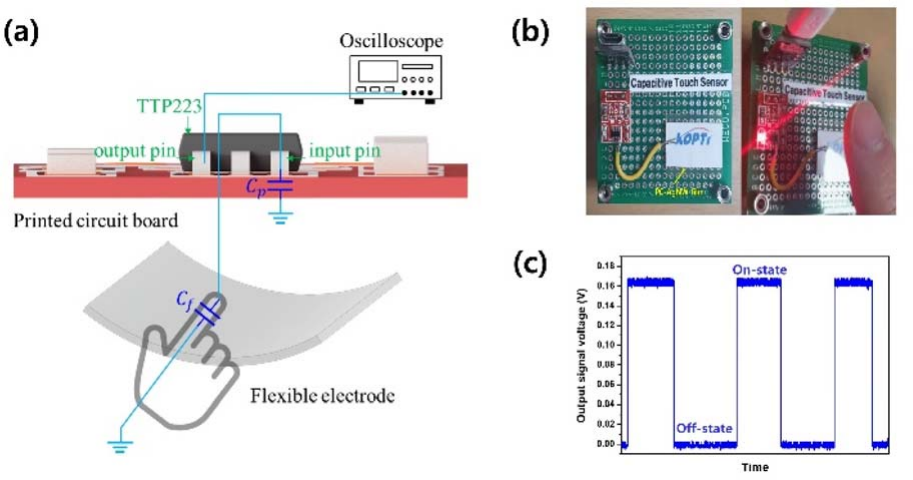
Demonstration of PC-AgNW-based capacitive touch sensor. (a) Working principle and (b) fabricated sensor. (c) Switching response with and without finger touch.

To apply our flexible electrode to a capacitive touch sensor, we integrated the fabricated PC-AgNW with a touch detector integrated circuit (IC; TTP223, Tontek Design Technology, Taiwan). Fig. 10 shows the working principle of our sensor. The PC-AgNW was electrically connected to the signal input pin of the IC. In the normal state, the sensor detects parasitic capacitance (*C*_p_) including the capacitance of the photocurable resin, PC film, and the printed circuit board (PCB) as a reference. When a finger touches the flexible electrode, the presence of the finger produces a capacitive difference (*C*_f_) that the IC detects, and the output signal is changed. The capacitive changes of the sensor were measured using an oscilloscope (DSO-X-4024A, Keysight, USA) that was connected to a signal output pin of the IC. Fig. 10(c) shows the sensor response with and without finger touch.

Iridescence, also termed rainbow effect, can be seen on plastic films. This effect could be mitigated using an anti-iridescence coating; however, by contrast, it could be made severe by external environmental factors such as temperature and UV exposure because of a thickness change and/or the shape deformation of the films. The transition temperature of the PC film is around 147 °C, much higher than that of the PET film (67–81 °C). Furthermore, PC films are well known UV-resistant plastics.^25^ These suggest that PC-based touch sensors are preferable than PET-based touch sensors for futuristic cars in terms of material stability.

Besides, the versatility of the PC film, such as high impact strength, weatherability, and high toughness, makes it more suitable for futuristic automotive applications.^26,27^ By integrating the PC-AgNW with the surface of a car's interior, general interior material can be converted into high value-added electronic components. Specifically, through an injection in-mold labeling (IML) technique,^28^ 3D-shaped component fabrication for car interior and electrode formation on the components can be simultaneously performed. Furthermore, interior components with the PC-AgNW sensor can be mass produced by the IML technique, indicating the potential of the PC-AgNW for commercialization.

Recent advances in nanostructure-based sensor system have been widely reported. Co-MoS_2_ nanomaterials on carbon electrode was fabricated for 4-aminophenol sensor,^29^ WO_3_ nanostructures was synthesized to detect H_2_S gas,^30^ and two-dimensional MXene were used for solar energy harvesting.^31^ The large-scale transfer of these various nanostructures will be included in future studies using our roll-to-roll UV lamination process.

## Conclusions

This paper introduced a roll-to-roll UV lamination process capable of large-scale AgNW transfer from a PET film to a PC film. We utilized an acrylate-based UV curable resin as an adhesion layer between AgNWs and PC substrate to rapidly fabricate flexible electrode with strong adhesion property. Systematic experiments were conducted to determine the optimal fabrication parameters with respect to the UV exposure time, lamination pressure, and laser dicing conditions. The determined parameters were 60 s of exposure time (120 mJ cm^−2^ of exposure energy), 69.67 gf cm^−2^ lamination pressure, 0.45 W laser dicing power, and 5 mm s^−1^ movable speed. At these optimal fabrication conditions, the sheet resistance of the PC-AgNW was as small as 36.79 Ω sq^−1^; in other words, the deviation was only 3.17% compared with the sheet resistance of the PET-AgNW. The optical transmittance of the PC-AgNW was more than 88% over the visible range, and it was improved compared with that of the PET-AgNW. The sheet resistance was almost constant after 50 taping and peeling cycles, which showed appreciable adhesion enhancement. Furthermore, the integration of a capacitive sensor with the PC-AgNW is demonstrated, and its feasibility as a touch sensor is shown.

The proposed roll-to-roll lamination process can be used to easily form large-scale electrodes on various versatile materials such as PC films, with improved adhesion to the substrate. It can be expected that the use of PC-AgNW and the IML technique can help increase the degree of freedom in the design of vehicle electronic components and facilitate the conversion of general interior parts of cars into aesthetic electronic machines.

## Author contributions

The manuscript was written through contributions of all authors. All authors have given approval to the final version of the manuscript.

## Conflicts of interest

There are no conflicts to declare.

## Supplementary Material
